# Emotions as pragmatic and epistemic actions

**DOI:** 10.3389/fpsyg.2015.01593

**Published:** 2015-10-26

**Authors:** Wendy Wilutzky

**Affiliations:** Institute of Cognitive Science, Universität OsnabrückOsnabrück, Germany

**Keywords:** emotions, action, philosophy of emotions, philosophy of mind, cognitive science

## Abstract

This paper explores the idea that emotions in social contexts and their intentionality may be conceived of as pragmatic or epistemic actions. That is, emotions are often aimed at achieving certain goals within a social context, so that they resemble pragmatic actions; and in other cases emotions can be plausibly construed as acts of probing the social environment so as to extract or uncover important information, thus complying with the functions of epistemic actions (cf. Kirsh and Maglio, 1994). This view of emotions stands at odds with the wide-held conception that emotions' intentionality can be cashed out in terms of representations of value. On such a position, emotions' intentionality has only a mind-to-world direction of fit while any world-to-mind direction of fit is deemed secondary or is even outrightly denied. However, acknowledging that emotions (qua actions) also have a world-to-mind direction fit has several advantages over the typical rendition of emotions as representations of value, such as accounting for emotions' sensitivity to contextual factors, variations in emotion expression and, importantly, assessing the appropriateness of emotional reactions. To substantiate this claim, several cases of emotions in social contexts are discussed, as the social dimension of emotions highlights that emotions are inherently ways of interacting with one's social environment. In sum, the construal of emotions in social contexts as pragmatic or epistemic actions yields a more fine-grained and accurate understanding of emotions' intentionality and their roles in social contexts than the insistence on a purely mind-to-world direction of fit.

## Introduction

Emotions are naturally social phenomena. Not only do emotions most commonly occur in social contexts, but they are mostly reactions to other people (Parkinson, 1995; Clark et al., 1996, p. 247), are expressed toward other people (Buss, 1992; Baumeister et al., 1994; Fischer and Roseman, 2007) and regulated by relations with other people (Brody and Hall, 2008). Further, a social setting is necessary for several emotions to arise in the first place, suggesting an inherently social quality of emotions. For instance, shame, envy, guilt, embarrassment, contempt, love, and hatred all require third parties as causes, targets, or observers in order for these emotions to occur in the first place. Thus, studying emotions in social contexts appears vital to understanding the nature of emotions and any adequate concept of emotions should be applicable to social contexts.

Surprisingly, the dependence of emotions on social contexts is often neglected in emotion research. On the empirical front, emotions are often studied in a laboratory setting by measuring subjects' reactions to pictures of stimuli such as spiders or snakes (Fischer and van Kleef, 2010), and, on the theoretical front, emotions are usually construed as representations of an object's value in some form or another (de Sousa, 2004, p. 61). While in the philosophy of emotions these representations may come in form of judgments of value and importance (Nussbaum, 2004, p. 183), reactions to known values (Mulligan, 2009) or attitudes toward an object exhibiting certain evaluative properties (Deonna and Teroni, 2012, p. 76), psychologists regard an emotion as the process of appraising a stimulus' significance to a subject, either according to various aspects of the stimulus' properties (such as Scherer's, 2001, stimulus evaluation checks) or as the manifestation of a fixed set of “core relational themes” (Lazarus, 1991; Prinz, 2004). Seemingly all these approaches attempt to explain emotions independently of any social dimension to emotions. Rather, emotions' function to represent values is assumed to be more fundamental to an emotion than any social functions they might have, so that the social dimension of emotions is considered secondary or even negligible (Fischer and van Kleef, 2010).

Further, as representations of value, on both philosophical and psychological emotion theories emotions are regarded as cognitions, in the sense that their intentionality has a mind-to-world direction of fit rather than a world-to-mind direction of fit^1^. Sometimes emotions are even explicitly denied any world-to-mind direction of fit (Döring, 2007, p. 384; Mulligan, 2007, p. 210–211; Deonna and Teroni, 2012, p. 83). Although the construal of emotions as evaluations (qua mind-to-world directed cognitions) is often befitting, it disregards emotions' motivational nature that leads to action (Scarantino, 2014, p. 156), i.e., the world-to-mind directed aspect of emotions' intentionality.

In social contexts the world-to-mind direction of fit of emotions' intentionality can hardly be denied. It seems self-evident that emotions occurring in social environments are ways of responding to and interacting with other social members. Hereby the relation between a social stimulus and an individual is not unidirectional, as the insistence on a purely mind-to-world direction of fit would imply. Instead, since social interactions necessarily entail that two or more social members act on and influence one another, an emotional individual influences the very stimulus of his emotion during such interactions. Thus, an emotion occurring in a social context is not a private and internal state that is triggered by a static stimulus in an individual, as a mind-to-world directed evaluative representation would imply (Parkinson, 1995; Parkinson et al., 2005). Rather, since the emotional stimuli are other social members' behaviors or emotions, which are subject to ongoing change and can be influenced, the relationship between individual and stimulus is dynamic and bidirectional (Fischer and van Kleef, 2010). Hence, in addition to the social environment impressing itself on the individual—which constitutes the often mentioned mind-to-world directedness—, so too the individual acts on his environment through his emotion—suggesting a simultaneous world-to-mind directedness of emotions. Another feature of emotions' world-to-mind direction of fit that is best highlighted in social contexts is that emotions are not always responses to stimuli but can also initiate socio-emotional interactions. That is, emotions in social contexts are not merely re-actions to stimuli but often also actions on others. Given the pronounced world-to-mind directedness of emotions in social contexts, construing emotions essentially or only as representations of value appears inadequate.

The aim of this paper is to demonstrate emotions' world-to-mind directedness and to show how it contributes to understanding emotions in social contexts. It shall become evident that acknowledging the world-to-mind direction of fit of emotions' intentionality has many explanatory advantages, such as accounting for context sensitivities, variations in emotion expression or the assessment of an emotion's appropriateness. To highlight the world-to-mind direction of fit of emotions' intentionality, the main concern will be to construe emotions as forms of action. Although the idea that emotions are action-oriented in nature is itself not new (cf., e.g., Scarantino, 2014), this article attempts to provide a novel insight into the active dimension of emotions by analogizing emotions to two kinds of action that have been distinguished in a different context by Kirsh and Maglio (1994): pragmatic and epistemic actions. Pragmatic actions are defined as transformations of the physical or social space in order to achieve a certain goal state in the world (ibid, p. 515), which amounts to that what is usually associated with the term “action.” Kirsh and Maglio's more innovative contribution to cognitive science is the identification of so-called *epistemic actions*. These are defined as physical actions that uncover information which is hidden or hard to compute mentally (ibid, pp. 513-4). Epistemic actions have the goal to change a system's computational state rather than the state of the world. As will be shown, emotions in social contexts often perform exactly those functions defined for pragmatic and epistemic actions. That is, emotions are often aimed at achieving certain goals within a social context, meaning that they resemble pragmatic actions, and in other cases emotions can be plausibly construed as acts of probing the social environment in order to uncover important information, thus complying with the functions of epistemic actions. The construal of emotions as either pragmatic or epistemic actions will reveal that the identification of emotions with representations of value, and therefore as mental states with only a mind-to-world direction of fit, is ill-fitting (pun intended) to adequately capture the intentionality of emotions. As forms of actions, emotions are consequently not only evaluative representations of a state of affairs in the world, but also comprise a directive aspect in their intentionality, so that emotions are best understood as active engagements with one's social environment. The extent to which these contributions matter for emotion theories will become evident during the discussion of possible objections to the proposed rendition of emotions as actions.

## Emotions as pragmatic actions

One indication that a mind-to-world directedness of emotions' intentionality alone is insufficient to account for emotions arising in social situations, is given by the significantly different ways in which a social context determines an individual's emotional reactions. That is, not only the type of emotion but also the extent to and way in which an emotion is expressed appears to depend on factors of the social situation in which the emotion occurs. Sadness and guilt, for example, predominantly occur when the emotional individual is among people he or she is intimate with, while it is rarely observed when an individual is among strangers (Buss, 1992; Baumeister et al., 1994). Similarly, joy is preponderantly expressed when the emotional individual is among affiliates and people with whom she wishes to consolidate her affiliative bonds (Parkinson et al., 2005, p. 162; Griffiths and Scarantino, 2009). Conversely, angry individuals tend to express their anger mostly toward people of lower social status, especially when the individuals think they can correct the behavior of the other person and have power or control over the other (Fischer and Roseman, 2007, p. 104). In these examples the idea that emotions are evaluations of objects or events and hence have only a mind-to-world direction of fit is difficult to fathom: For instance, it is not apparent why a particular object or event would be ascribed those evaluative properties that correspond with sadness when one is among friends but not when one is among strangers. The same holds for joy. Likewise, what relative social status an individual has should not impact the evaluation of a stimulus *per se*, so that the stimulus' evaluation leads to anger in one case but not the other. Instead, the close relation between the occurrent emotions and the nature of the social contexts in which they arise rather reflect an important function of emotions on the interpersonal level, namely to influence social relations according to an interpersonal motive (Fischer and Manstead, 2008, p. 458). Hence, the observable context dependencies are not only the consequence of an object's represented evaluative properties, but the result of the difference in afforded possibilities for interacting with or acting on one's environment, thus indicating a world-to-mind direction of fit.

Diverging from most psychological and philosophical emotion theories that regard emotions essentially as representations of value, social psychologists often study emotions in terms of the functions they perform in social contexts (cf, e.g., Parkinson, 1995; Clark et al., 1996; Parkinson et al., 2005; Fischer and Manstead, 2008). That is, emotions are considered to be ways of achieving social goals by configuring or altering one's standing and one's relations within a social group (Fischer and Manstead, 2008, p. 457). Sadness, for example, is a sign to others that one is vulnerable and in need of support, thereby serving an affiliative function, especially when crying is involved. In contrast, fear aims at putting distance between oneself and others, thereby serving a distancing function. Anger, in turn, serves the purpose of imposing change upon another person's attitude or behavior, so that, e.g., a threatening gesture to someone can make that someone back off or telling off a friend because he is late for an appointment is meant to make him not be late again (ibid, p. 458).

Explicating emotions in terms of their functions implies that emotions are ways of attaining certain goals or bringing about certain effects in an individual's social environment, i.e., they are world-to-mind directed in their intentionality. This description of emotions coincides neatly with the definition of pragmatic actions offered by Kirsh and Maglio (1994, p. 515): transformations in a physical or social space to advance toward a certain goal state. The construal of emotions as pragmatic actions offers a plausible and significantly different perspective on emotions in social situations than the typical contention that emotions are essentially evaluations of objects or events. It further provides straightforward explanations of emotion processes where the typical characterization of emotion fails or at least has some difficulty to do so. This shall become evident in the following discussion of examples. In particular, the proposal that emotions are pragmatic actions will be substantiated by showing that emotions may be seen as pragmatic actions with either short- or long-term goals. That is, the goal state which pragmatic actions are aimed at can either be an immediate effect of a pragmatic action or lie at the end of a series of pragmatic actions (ibid.), and emotions in social contexts too can serve either immediate pragmatic purposes or they can be elements in a long-term strategy of relationship configuration.

### Emotions as pragmatic actions with short-term goals

Already above the descriptions of some social functions of emotions revealed that the emotion which an individual exhibits in a given social context can yield certain behavioral effects from other social members. For example, an individual can try to get needed support by exhibiting sadness or change the behavior of his vis-à-vis through anger. Here emotions are direct functions of what an emotional individual can achieve with that emotion in the social situation. An exemplary study of such direct effects yielded through emotions is one by Stein et al. (1993), where subjects were asked to describe situations in which they had become angry. It was found that whether a perceived loss elicits anger or sadness depended on subjects' prospects of obtaining compensation in the remembered situation. These findings are inexplicable if anger were merely the evaluation that one has been wronged, but intelligible if anger is seen as a strategy to elicit a certain reaction in others. Viewing emotions as strategies of relationship configuration (Parkinson, 1995, p. 295), explains why an individual should become upset in one way and not another: anger and sadness serve very different social goals and an emotional individual will react with that behavioral strategy which is most likely to achieve the desired goal.

The pragmatic function of an emotion may vary within one and the same emotion kind, so that there is not necessarily a one-to-one mapping from emotion kind to social function. To see this, consider an aggressive reaction vs. sulking in response to an insult. According to most emotion theories, both are manifestations of anger, since in both cases an event is evaluated as an offense, or, according to appraisal theories, as motivationally incongruent. But although the evaluation may be the same in either case, all other aspects of the emotion (e.g., expression, feeling, physiological responses) differ vastly. So, a reference to the evaluative content alone cannot explain why anger should manifest itself in such very different ways. When construed as pragmatic actions though, a distinctive difference between aggression and sulking quickly becomes apparent: Whereas, the former is a brusque attempt to interrupt the happenings of a situation, the latter is a denial of all social transactions until appropriate concessions are obtained (Griffiths and Scarantino, 2009, p. 440). Strategic concerns (e.g., whether or not compensation can be obtained) or the effects of and on one's social standing (e.g., is the behavior compatible with one's social standing or will it cause others to regard one in a lower social status) will play a decisive role in determining how an individual will react emotionally. These aspects are missed if emotions are equated with evaluative representations only. Acknowledging that emotions are aimed at certain social goals therefore makes more aspects of emotions and their intentionality intelligible, than when they are reduced to evaluative representations with only a mind-to-world direction of fit.

Another example of an immediate purpose emotions may serve is their communicative function which was studied in an experimental set-up devised to investigate embarrassment (Leary et al., 1996). In this study, subjects were asked to record a karaoke-style performance of a notoriously cheesy love-song under two different conditions: In one condition, the experimenter who recorded the performance signaled to the participant that he was aware of the embarrassing nature of the task and had registered their discomfort; in the other, the experimenter made no such sympathetic indications. The recordings of participants' performances as well as a subsequently administered questionnaire were analyzed to determine the levels of the participants' expressed and experienced embarrassment over the task. The data revealed that subjects both showed and reported less embarrassment in the first condition, where they were given reason to believe the experimenter sympathized with them, than in the second condition. Since the target object remained identical in the two conditions (participants were given the same task to perform), the researchers concluded that, rather than only reaction to their evaluation of the task, the participants' embarrassment functioned as means to communicate to the experimenter their low opinion of the song they were singing and their desire to conform to community standards. In other words, the interpretation of the findings offered by the study's authors suggests that emotions have a pragmatic nature and hence a world-to-mind direction of fit.

Granted, the results of this study do not necessarily rule out the possibility that emotions are representations of value. Arguably, participants' evaluations of the situation may have been contextually richer than merely the representation of the task's properties in isolation. For instance, whether or not participants were aware of the experimenter's knowledge of their embarrassment for performing the task may have been included in their evaluative representation of the situation. That is, the observed variance in embarrassment could have been the result of a difference in the way the situation was represented after all. Yet, albeit not an outright refutation of the construal of emotions as evaluative representations, the study's findings are much more easily and more parsimoniously explained by ascribing a communicative function to emotions. To see this, consider how the interpretation offered by Leary and colleagues immediately addresses the expressive aspects of an emotion and the effects these may have on a situation. In the explication of emotions as pragmatic actions in social contexts these aspects come as part and parcel of an emotion, since it is through the expression of an emotion that an individual interacts with her social environment and achieves a desired change in the social space. In contrast, the construal of emotions as evaluative representations requires a separate account of how an evaluative representation leads to a certain expression of the emotion, while the effects of the expression are seemingly ignored completely. Thus, given the advantage of a more comprehensive while simultaneously also parsimonious account, viewing emotions as pragmatic actions and thereby having a world-to-mind direction of fit appears to be the more favorable explanation of this experiment's data than the idea that emotions are evaluative representations with a strictly mind-to-world direction of fit.

### Emotions as pragmatic actions with long-term goals

Aside from the just portrayed immediate effects of emotions in social situations, emotions can also serve to achieve long-term goals for an individual's social relations. Similarly as in the cases just described, where exhibiting one emotion instead of another aims at a certain short-term goal, long-term effects of emotions on social relations can be achieved by the same means. Illustrating this is a study on anger vs. contempt, in which these sometimes conflated emotions are characterized as decisively different, not only in terms of their expressions and physiological characteristics, but especially in their social functions (Fischer and Roseman, 2007). The authors of the study found that whether subjects react to someone's action with anger or contempt involves more than finding the other blameworthy or perceiving an offense in the other's behavior (i.e., the evaluation of that behavior with only a mind-to-world directed content). Whether subjects reacted with contempt or anger correlated significantly with the desired long-term effects that emotion would have on the interpersonal relationship in question: Whereas anger is characterized by a short-term attack response but has the effect of long-term reconciliation, contempt is characterized by rejection and social exclusion in both the short and long term (ibid, p. 103). Thus, again, the difference in the emotional reaction cannot be the result of the evaluation of a presented stimulus alone, but must rather be ascribed to pragmatic factors in the subjects' strategies for long-term relationship configuration.

On a different note, consider those emotions that are intrinsically long-term phenomena, such as love, hate, grief, resentment, or jealousy. These emotions necessarily persist over a longer period of time. It would, for instance, be questionable to claim that a person loved someone, but did so only for the duration of a few minutes. This may be infatuation, interest, or a crush, but, as the late Solomon argued (Solomon, 2007, p. 194), love needs to be cultivated and is a process which consists in several choices and actions made over a longer course of time. This rich notion of love conforms with the folk-psychological concept of love, and, arguably, any viable emotion theory should account for it, if the theory's explanandum is to resemble that which any lay person understands by the term instead of some artificially constructed concept. But this notion of love has proven difficult to square with many emotion theories. Especially for psychological emotion theories the question how to accommodate long-term emotions into their theoretical framework poses a problem, since here emotions are often explicitly defined as short-lived episodes (cf., Scherer, 2001). Some proposals of what long-term emotions such as love are, include mental attitudes toward certain objects (Deonna and Teroni, 2012) or dispositions to undergo particular kinds of short-lived emotion episodes (Scherer, 2001). However, these attempts at making long-term emotions intelligible are unsatisfactory on two fronts: First, they treat long-term emotions as a different kind of phenomenon than short-term emotions, since long-term emotions are ascribed to a different ontological category. As pointed out above, such a stark deviation from the folk-psychological concepts of emotions, where long-term emotions such as love and hate are equally commonplace examples of emotions as fear or anger, runs the risk of changing the explanandum of emotion theories to some artificial construct. Second, from the equation of a long-term emotion with an attitude or disposition to undergo certain emotion episodes, it follows that an individual may have a long-term emotion without that emotion necessarily ever having to be instantiated. That is, it is possible for person A to love person B, meaning that A has the disposition to undergo certain emotion episodes upon meeting B, but A may not ever meet B. So, although A may have the disposition to love B, A may never actually experience an episode in which that love is instantiated. At the very least, this possibility of loving someone, in the sense that one has a disposition or attitude of a certain kind, but without ever experiencing or instantiating that love is very odd. It remains to be concluded that many standard emotion theories have difficulties accounting for long-term emotions.

In contrast, when explicating social emotions as ways of relationship configuration it becomes obvious that the object of study can be itself a long-term phenomenon, namely the interpersonal relationship and the changes it undergoes. Moreover, when emotions in social contexts are explicated as kinds of pragmatic actions on an interpersonal relation, this directly opens up the possibility to construe long-term emotions as a series of individual actions which are all aimed at one particular goal of relationship configuration. This is entailed by Kirsh and Maglio's definition of pragmatic actions, where the goal of the action can also lie at the end of a series of actions (Kirsh and Maglio, 1994, p. 515). Just like the act of getting a beer from the fridge may consist in several individual steps (like getting up, walking into the kitchen, opening the fridge etc.), the act of loving someone may consist in a number of emotional episodes (like being happy upon seeing them, sad when they leave, afraid when they are in danger etc.). This possibility to account for long-term emotions with the same explanatory tool as for short-term emotions, i.e., as goal-directed actions, provides a compellingly parsimonious account of emotions. Also, the idea that an emotion is aimed at a long-term goal of relationship configuration and every action toward this long-term goal therefore constitutes that one and the same emotion, bars the odd possibility of having a disposition or attitude of a long-term emotion, without ever experiencing that emotion, as discussed above. Furthermore, the idea that single emotion episodes constitute an overarching long-term emotion just as single actions constitute a complex action, offers a phenomenologically plausible description of the relation between a long-term emotion and the single emotion episodes that constitute it. That is, a long-term emotion such as love or hate or jealousy manifests itself and is experienced in the single emotion episodes constituting it, such as the sadness over a loved one's departure, the joy over one's enemy's downfall or the anger over an adversary's advances. These individual emotion episodes are not prompted by the long-term emotion, in the sense that they are effects of a distinct cause, but the long-term emotion simply consists in these single emotion episodes and are also experienced as such^2^. Relatedly, Helm (2002, p. 22) describes how a mode of caring results in a rational pattern of emotions with a common focus, so that certain ways of caring commit one to a set of emotions, feelings and desires in situations concerning the focus. Caring about someone or something therefore simply is to have certain emotional experiences toward that object.

In sum, the construal of short- and long-term emotions as pragmatic actions offers a significantly different perspective on emotions from the approach of most emotion theories to view emotions as mind-to-world directed evaluative representations. As shown, by taking into consideration how emotions are aimed at goal states in social contexts, instances of both short- and long-term emotions can be made intelligible which the usual approaches have difficulties accounting for or can give only cumbersome explanations. Also, not only did treating emotions as pragmatic actions in social contexts produce parsimonious explanations in the individually discussed cases, but, overall, the fact that both short- and long-term emotions can be fittingly described with one and the same explanatory tool yields a concise and sparse account of emotions on varying time-frames—something the standard construal of emotions as evaluative representations founders on. Finally, the idea that single emotion episodes are all part of one overarching long-term emotion, just like single pragmatic acts can be part of one complex action, seems to be in accord with the general phenomenality of such emotional occurrences.

## Emotions as epistemic actions

The unique contribution which Kirsh and Maglio's article (Kirsh and Maglio, 1994) made to cognitive science, and which has thus far not been related to the philosophy of emotions, is the insight that not all actions are necessarily pragmatic in their function. That is, some actions, rather than being directly aimed at changing the world so that it conforms to a particular goal state, are performed to uncover information that is hidden or to simplify an agent's problem-solving task. These actions serve an epistemic function instead of a pragmatic one (ibid, p. 513). As an example of epistemic actions Kirsh and Maglio focus primarily on Tetris-players' habit to physically rotate the appearing blocks to determine where they would best be placed, instead of performing this rotation mentally. Other examples include gathering information about the environment through exploration, like when scouting unfamiliar terrain in order to determine where to set up camp, or using one's environment to simplify a cognitive task, like when arranging mechanical pieces in a particular order to determine how to assemble them (ibid, p. 515). All these are “ways an agent has of modifying the external environment to provide crucial bits of information just when they are needed most” (ibid, p. 542). Importantly though, the changes made in the environment in order to achieve the desired epistemic state are not the goal of the epistemic action. This is the critical point in which epistemic actions differ from pragmatic actions: Unlike pragmatic actions, epistemic actions are not aimed at changing the world to be a certain way, but are aimed at achieving an epistemic state for the cognitive system (ibid, p. 514).

This places epistemic actions in an odd place between world-to-mind and mind-to-world direction of fit: On the one hand, they are actions, meaning they are performed transformations of physical or social space in order to achieve a certain goal or purpose. As actions they (should) have a world-to-mind direction of fit. Yet, unlike pragmatic actions, the goal of epistemic actions does not lie in bringing about a change in the world, but rather in altering one's epistemic state. That is, epistemic actions are not world-to-mind directed in the sense that they are aimed at bringing about a particular state of the world that ought to obtain. On the other hand, epistemic actions result in epistemic states, which are mind-to-world directed. However, epistemic actions do not have a mind-to-world direction of fit either because, albeit they disclose facts about the world, they do not, strictly speaking, represent these. Yet, despite the difficulty to classify epistemic actions using the typical categories of intentional states' direction of fit, they should nonetheless be regarded as intentional states of some kind, since they are skilled or intelligent forms of interacting with the world. Rather than having neither direction of fit, epistemic actions seem to have both^3^.

What might shed some light on the nature of epistemic actions' intentionality concerning the conundrum about their direction of fit, is to frame the matter in terms of their fulfillment and correctness conditions: Since epistemic actions aim at providing facts, it appears to be their fulfillment condition to establish the truth values and correctness conditions of a state of affairs in the world. In other words, epistemic actions are world-to-mind directed processes aimed at bringing about mind-to-world directed states.

The unique intentional nature of epistemic actions can be used to capture aspects of emotions' intentionality in social interactions that are often overlooked. For, emotional interactions with others too can be understood as probing for certain information that becomes central to the unfolding of an emotion process. This probing does not necessarily require a premeditated hypothesis that is deliberately tested, but can be a very simple behavioral mechanism similar to ones observed in animals. A suitable example is provided by the ethologist Hinde's (1985) observation of birds issuing threat responses toward approaching opponents. The threat signal bears all characteristics of anger, yet in most cases the birds flee after issuing it. Statistically, the issued threat signal is thus rather an indication of a fear reaction. To resolve this bifurcation, Hinde proposes that the bird's issued threat response is rather a way of testing its opponent's intentions and power, i.e., the bird probes whether its rival is at all interested in attacking or whether he can easily be made to back off. Likewise, dogs often bark fiercely at an approaching opponent, i.e., display anger, only to demonstrate submissiveness shortly afterwards if their opponent proves to be more dominant. In both examples, the initial emotional reaction is a way for the animal to probe the situational conditions and the gathered information helps determine its future actions. In other words, the animal performs an epistemic action and from the newly attained epistemic state, its subsequent emotional reactions ensue. Importantly, the further development of the emotion process depends on the information acquired through the epistemic action (e.g., if the bird discovered that his threats were effective, it would not flee in fear but continue with its display of anger, or the dog would not cease to display threats if they proved effective in intimidating its opponent).

Analogous behaviors can be observed in humans' interactions with one another. Continuing with the example of anger, an individual may, after receiving a possibly threatening cue from another person, ask somewhat aggressively, “What's that?” or “What do you want?,” with the corresponding posture (e.g., raised chin, protruded chest), facial expression (e.g., narrowed eyes, tight jaw) or gesture. This emotional signal serves to retrieve more information about the other's intentions and the situation, i.e., it has an epistemic function. Although the individual who reacts in this way is signaling a readiness to respond angrily just in case the other's action was in fact meant as an offense, the development of the emotion process remains to be determined and depends fully on the other's “reply” to one's “question.” That is, depending on the information that is retrieved by one's probing action, one may immediately cease to be angry (e.g., if the other were to clarify that he had no offending intentions in mind), become afraid (e.g., if the other were to respond even more forcefully) or the anger may persist (e.g., if the other were to continue with his behavior). Further examples of human probing behavior are pouting, teasing, sulking, and sometimes also smiling. In each of these conducts emotions like sadness, contempt, anger, and joy, respectively, are employed to probe one's social environment, i.e., to test another person's reaction and see what possibilities the situation affords. Relatedly, Griffiths and Scarantino (2009) portray emotions in social contexts as possible forms of negotiations, an idea they adapt from Hinde's work on emotion expression in animals and humans. Although emotional behaviors can be expressions of an inner psychological state, often times emotional behavior is rather aimed at gathering feedback from one's social environment. In the latter case, the emotional signals produced by an individual are intentionally ambiguous, so that the outcome of the social transaction remains open ended and depends crucially on the recipient's response (ibid, p. 446). Many emotional signals that are sent in social interactions are hence aimed at uncovering information and, therefore, arguably epistemic actions.

Clearly, this rendition of emotions operating as epistemic actions runs contrary to the idea that emotions are representations of value, which would require an already established evaluation of the situation. Yet, from the fact that an evaluation is not yet fixed when the social environment is probed with an emotion, it must not follow that these instances of emotion are devoid of all value-related content. For, even though the situation's value may not yet be established, the emotion qua epistemic action is aimed at discovering the correct evaluation of the situation and thereby still concerns the situation's value. Such probing for the evaluative quality of a social stimulus by actively engaging with it bears several advantages over the view that the evaluation of a social stimulus is determinately fixed at the outset of an emotion process. First, it simply requires less cognitive capacities to probe for information at that moment when it is needed, than to acquire and represent all details in advance or to arrive at the relevant information through costly internal computations (Noë, 2004; Clark, 2008). Furthermore, seeing that the objects which emotions in social contexts are directed at are other people, the objects of emotions are subject to ongoing change. Thus, in order for emotional interactions to be successful the information they rely on must be continuously updated. Often the temporal dynamics of social interactions and emotions transpire on the scale of milliseconds, so that an immediate uptake of information is crucial. For example, a fleeting facial expression may matter significantly to the unfolding of an interaction between two people (Ekman, 2003). Finally, epistemic actions can be performed repeatedly, so that a cognizer's epistemic state is continually updated (like repeatedly touching an object that is being heated in order to determine whether it has reached the desired temperature yet). In the case of emotions, this allows for a fine-tuned specification of a situation's or event's evaluative nature and minimizes errors in evaluative assessments. In sum, continuously probing for or otherwise exploring a situation's meaning or another person's intent through epistemic actions is not only an efficient and computationally undemanding way of making information available to cognitive processes like emotions, but, because the acquired information is immediate and continually updated, it is also bound to yield very accurate assessments and successful emotional interactions. Standard emotion theories miss out on these advantages by ignoring the dynamic nature of emotion processes and insisting instead that an evaluative representation of a situation must be fully established first in order for an emotion to occur.

It is worth contemplating further the importance of probing for the correct evaluation of a situation and allowing for an emotion to develop with every acquired information-update. It is these dynamic features of emotion processes which enable the richness, complexity, and fineness of our social interactions. If it were necessary for an evaluation of a situation to be fully established and settled on at the outset of an emotion process already, thus barring the possibility of making further inquiries and amending one's evaluation accordingly, this would leave very little room for fine social interactions and lead to rather primitive behaviors instead. In fact, a lack of explorative abilities and flexibility in emotional processes characterizes many affective disorders, such as anger or anxiety disorders, problems with anger management, phobias, and mania. It also undermines the possibility of being attuned to one's social concomitants' emotions and thereby prohibits important emotional mechanisms in social conduct, like empathy. Hence, it is a significant feature of social and emotional intelligence not to be too rigid in emotional interactions, but rather allow for evaluations to be searched for or inquired after, thus leaving room for emotions to unfold dynamically. Yet, acknowledging the importance of an adaptable evaluative content that is susceptible to new information clashes with the typically discussed themes in cognitive emotion theories, which comprise the irrationality of emotions and emotions' resistance to judgments or beliefs.

Recognizing that emotions are first and foremost social phenomena and embedded in the social contexts in which they arise debunks the understanding of emotions as singular and detached representations of an event's evaluative properties, which, once cast, remain unchanged (Fischer and van Kleef, 2010, p. 209). Rather, a system of reciprocal causation or dynamic coupling is created between an individual and his social environment through ongoing emotional interaction (Griffiths and Scarantino, 2009, p. 445–446). Emotions may be understood as means by which an individual can interact with her social environment, and this bidirectional nature of emotions' intentional structure in social contexts is fittingly captured by epistemic actions, as is their adaptable evaluative content.

## Anticipation of objections and discussion

It is to be expected that the made proposal to construe emotions as pragmatic or epistemic actions will run into opposition at several points. This section aims at anticipating three such objections and to provide counterarguments and clarifications in order to avert these. In doing so, the consequences for ongoing theorizing about emotions shall become evident.

One objection to be expected is that the described cases of emotions as pragmatic or epistemic actions are not genuine emotions. That is, although it may be granted that the performed actions in the given examples are aimed at achieving certain pragmatic or epistemic goals, the described behaviors are only pretenses of emotions to reach that goal and not sincere emotional reactions to the situation's evaluative properties. Especially the referral to emotions as strategies may imply that feigning an emotion is simply part of an agent's deliberate plan.

However, the purposefulness of emotions must not be the product of a premeditated and deliberate strategy. In both the renditions of emotions as epistemic and pragmatic actions, the emotions are immediate and intuitive reactions to the social dynamics of a situation, for some of which there exist homologous animal behaviors. The social dimension is a fundamental element in the emotion process, so that the social effects of emotions are available to individuals without involving any premeditated planning (Fischer and van Kleef, 2010, p. 208–209). Thus, the observed emotional reactions are rather the effects of very basic and inherent processes rather than deliberate and intentional plans. Relatedly, as Griffiths (2004) has argued, emotions track possibilities for interactions. That is, emotions can simultaneously be the assessment of a situation's meaning as well as an intention to act a certain way. This is so because the significance of a stimulus situation is not only evaluated in terms of what has happened, but also in terms of what will happen if the emotion is produced. Hence, an intention to act is an inherent part of an emotion's evaluative intentionality and not a derivation from a separate evaluative representation. In sum, the fact that emotions can be purposeful social interactions need not imply that they are deliberate pretenses.

A related objection may be that the discussed examples are not genuine instances of emotions but only expressions of emotions or other forms of bodily reactions. Concerning those emotions that were presented as pragmatic actions, it may be called into question whether the behavior that serves the pragmatic function really is part of the emotion itself and not just a behavior caused by the emotion. In other words, the difference in observed expressions of emotion in the discussed cases are just that: differences in expression, but not necessarily differences in emotion. Similarly, the epistemic actions that are performed to probe for a situation's value may have the appearance of emotional behaviors, but are not actually emotions. On this objection, both in the case of pragmatic and epistemic actions, the bodily behavior or expression which may look like an emotion, is considered to be clearly distinct from the actual emotion itself. Such a segregation of the bodily and cognitive aspects of emotions is often found in philosophical cognitivist position on emotions (cf., e.g., Solomon, 1976; Nussbaum, 2004; Döring, 2007), where the evaluative content of an emotion is regarded as the defining feature of an emotion, while the bodily aspects are considered secondary or even negligible.

Clearly, this objection is a disagreement over how to define the term “emotion.” While many (mostly philosophical) theories presuppose a certain intentional structure with an evaluative content as the defining mark of emotions, there are also strong arguments in favor of the position that behaviors which exhibit all essential physiological characteristics of emotions deserve to be called emotion. For instance, affect program or basic emotion theories, both of which stress the evolutionary continuity of emotions in a Darwinian tradition (e.g., Ekman, 2003), would regard the behavioral aspect as equally, if not even more, crucial to identifying emotions as the intentional structure. Also among contemporary appraisal theorists criteria such as motivational force, activation of somatovisceral and motor systems, as well as attentional and organismic control precedence are recognized as equally defining features of emotions as the appraisal of a stimulus (cf. Frijda and Scherer, 2009, p. 143–144)^4^. The idea of an emotion devoid of any physiological arousal or bodily expression and a biased insistence on a particular intentional structure as the sole definitive mark of emotions instead, appears to be a disjointed notion of philosophical cognitivism. Thus, such an approach leads to a scientifically isolated position. Moreover, as pointed out in the discussion of emotions as pragmatic actions, any approach that treats the emotion expression as separate from the emotion owes an additional explanation of how corresponding expressions of emotion are produced. An emotion theory which incorporates the expressive aspects of an emotion from the outset, therefore fares better with respect to parsimony and completeness. Lastly, the segregation of the emotion's expression from the emotion itself simply is phenomenologically implausible. That is, the bodily changes take place and are experienced throughout an emotion are just as much part and parcel of the intentional structure of an emotion as any other intentional content (cf. Goldie, 2000).

A third possible objection is that, even if the depicted emotional reactions were genuine, they would be incorrect or inappropriate because they misrepresent the situation's evaluative properties. It is a key feature of many emotion theories to assume that emotion types are individuated by their evaluative contents, so that each type of emotion corresponds to one certain evaluative content, either in form of a formal object, core relational theme or appraisal pattern (cf., e.g., de Sousa, 1987, p. 20; Prinz, 2004; Deonna and Teroni, 2012, p. 41). Anger, for instance, is individuated through the evaluative content of a slight or offense, a demeaning offense against me or what is mine, or a motivational incongruence for which another person is accountable, respectively, and every instance of anger must have this evaluative content, else it is incorrect and inappropriate (cf., e.g., de Sousa, 1987, p. 108; Mulligan, 2007, p. 209; Deonna and Teroni, 2012, p. 81). In the previous sections, the depicted emotions' types often did not correspond with their presupposed evaluative contents (in the case of emotions as pragmatic actions, e.g., whether anger or sadness arose depended on the emotional individual's possibilities for social interactions rather than the evaluation of the stimulus event as an offense) or they lacked a proper evaluative content altogether (in the case of emotions as epistemic actions the evaluation of the stimulus situation still remained to be determined). Therefore, it may be argued that these emotional reactions are incorrect and inappropriate for the depicted situations.

Yet, the idea that the correctness or appropriateness of an emotion can be assessed by whether or not it corresponds to a particular sort of evaluation is questionable. It stems from the presupposition that emotions are purely mind-to-world directed and thereby have corresponding correctness conditions. Conceding instead a world-to-mind direction of fit for emotions' intentionality immediately results in different assessments of the appropriateness of emotional reactions to stimulus situations—ones which often seem more fitting and desirable. In the case of emotions as pragmatic actions, for instance, recognizing the world-to-mind direction of fit of emotions entails that the appropriateness of the emotional reaction is assessed with regard to the emotion's pragmatic function. That is, whether or not a certain emotion qua pragmatic action is appropriate depends on whether the intended aim is appropriate and the means by which it is achieved are socially adequate (cf. D'Arms and Jacobson, 2000 or Wilutzky, submitted). Recall, for instance, that the social function of anger is to cause someone to change her behavior. Anger can be exhibited to this end without there necessarily having been an offense, e.g., when scolding a child for dangerous behavior or a football player's reaction when being tackled by a member of the rival team. In both situations the occurring anger serves an appropriate purpose, i.e., protecting the child from harm or intimidating the rival, and seems socially adequate for the situation. The emotional reaction is by no means incorrect or inappropriate. Rather, not exhibiting anger in these cases would appear to be inappropriate. Concerning emotions qua epistemic actions, as argued at the end of the discussion of emotions as epistemic actions, searching for the correct evaluation of a situation makes the richness and fineness of our social interactions possible. Probing for and exploring the correct evaluation of a situation is thus highly appropriate and failing to do so can result in inappropriate and even pathological behaviors. Hence, determining emotions' appropriateness with respect to their world-to-mind directedness instead of their mind-to-world direction of fit yields assessments of their entire, that is, also their moral and pragmatic, adequacy. In this light, the emotions described in the previous sections are arguably correct and appropriate ways of reacting to a situation.

One final clarification that may be in order, concerns the different time-scales on which emotions transpire. Whereas emotions were presented as long-term phenomena that may persist over years when discussing emotions qua pragmatic actions, it was pointed out that emotions may be quick and immediate reactions to a situation when discussing emotions qua epistemic actions. At first glance, these different temporal characterizations appear contradictory, and, indeed, there seems to be a related divide among emotion theories between those that regard emotions as possibly complex cognitive phenomena and those that study emotions as instantaneous basic and automatic reactions to stimuli. However, for the above discussion of emotions the variations in temporality do not necessarily present a conflict. To the contrary, the varying temporal scales on which emotions transpire underline the dynamic structure of emotional engagements with the world previously argued for. As became evident in the discussion of emotions as epistemic actions, an emotion process may encompass several iterative probings or other forms of repeated interaction with the environment. Likewise, it was shown that an emotion qua pragmatic action may either be aimed at an immediate goal of relationship configuration or a long-term one, which in turn may consist in several instances of emotions. Thus, an emotion may indeed be a quick and immediate reaction in the realm of milliseconds. Then, what is being referred to as “emotion” is one step in a possibly temporally extended and iterative emotional interaction. But emotions do not necessarily end after one single reaction to a stimulus, as commonplace construals of emotions as evaluative representations seem to imply. Since emotion processes can encompass several iterations of a dynamic exchange between an individual and her social environment, they may have a much longer duration in total. Both temporal orders in which the term emotion may be understood, that is, either as immediate or continuous actions, can be addressed when the concepts of pragmatic and epistemic actions are applied to emotions as suggested above^5^.

## Concluding summary

The aim of this paper was to challenge the usual construal of emotions' intentionality as evaluative representations with only a mind-to-world direction of fit and to promote instead the alternative view that emotions often also exhibit a world-to-mind direction of fit. To this end, different instances of emotions in social settings were discussed. It was shown how these can be construed as pragmatic or epistemic actions, and that such construals bear several advantages over the prevalent assumption that emotions may be equated with representations of value: Seeing emotions as pragmatic actions highlights the social functions emotions play in relationship configurations, which can make differences in emotional reactions intelligible that standard emotion theories have difficulties accounting for. Also, the construal of emotions as pragmatic actions often yields more parsimonious explanations of emotional processes, not least because it is easily applicable to both short- and long-term emotions. Understanding emotions as epistemic actions further highlights the dynamic nature of emotions, consisting in continuous interactions with the environment that allow emotions to be adaptable, accurate, and fine-tuned engagements with the world. Importantly, conceding that emotions can have a world-to-mind directedness by no means entails that these emotions are not genuine reactions to a situation or that they are inappropriate.

### Conflict of interest statement

The author declares that the research was conducted in the absence of any commercial or financial relationships that could be construed as a potential conflict of interest.

## Footnotes

^1^In this paper Velleman (1992, p. 8) definitions of the two directions of fit will be taken as a guideline. That is, in mind-world directed intentional states a proposition is “grasped as patterned after the world,” i.e., as something true, whereas in world-mind directed states a proposition is “grasped as a pattern for the world to follow,” i.e., regarded as something to be brought about or to be made true. The former are referred to as cognitions while the latter are termed conations.

^2^I am indebted to one of my reviewers for prompting these considerations, and also for pointing out that similar ideas were much more carefully already developed by Aristotle in his contemplations concerning the concept of hexis, i.e., our tendencies to form or shape our emotions through actions. For a succinct and comprehensive summary and discussion, see Slaby and Wüschner (2014).

^3^In this respect epistemic actions resemble Millikan's (1995) pushmi-pullyu representations, which are also both directive and descriptive but without being only the one or the other.

^4^Note that there are about as many different appraisal theories as there are theorists. However, there seems to have been a general tendency in the early appraisal theories, such as those of Magda Arnold and Richard Lazarus, to regard an emotion as the effect of an appraisal. That is, the bodily changes and action tendencies were regarded as the proper constituents of the emotion, while the cognitive appraisal was only a precursor to it. This indicates that in appraisal theories too the physiological aspects of emotions are indisputably part and parcel of an emotion process.

^5^These considerations were prompted by one of the many constructive comments of one of my reviewers.
